# Supplementary material for: Immune synapse formation promotes lipid peroxidation and MHC-I upregulation in licensed dendritic cells for efficient priming of CD8+ T cells
Source: Nat Commun. 2023 Oct 25;14:6772. doi: 10.1038/s41467-023-42480-3 (PMC10600134; doi:10.1038/s41467-023-42480-3)
Supplement: Supplementary file 1 — Supplementary Information [file 41467_2023_42480_MOESM1_ESM.pdf]

Figure S1

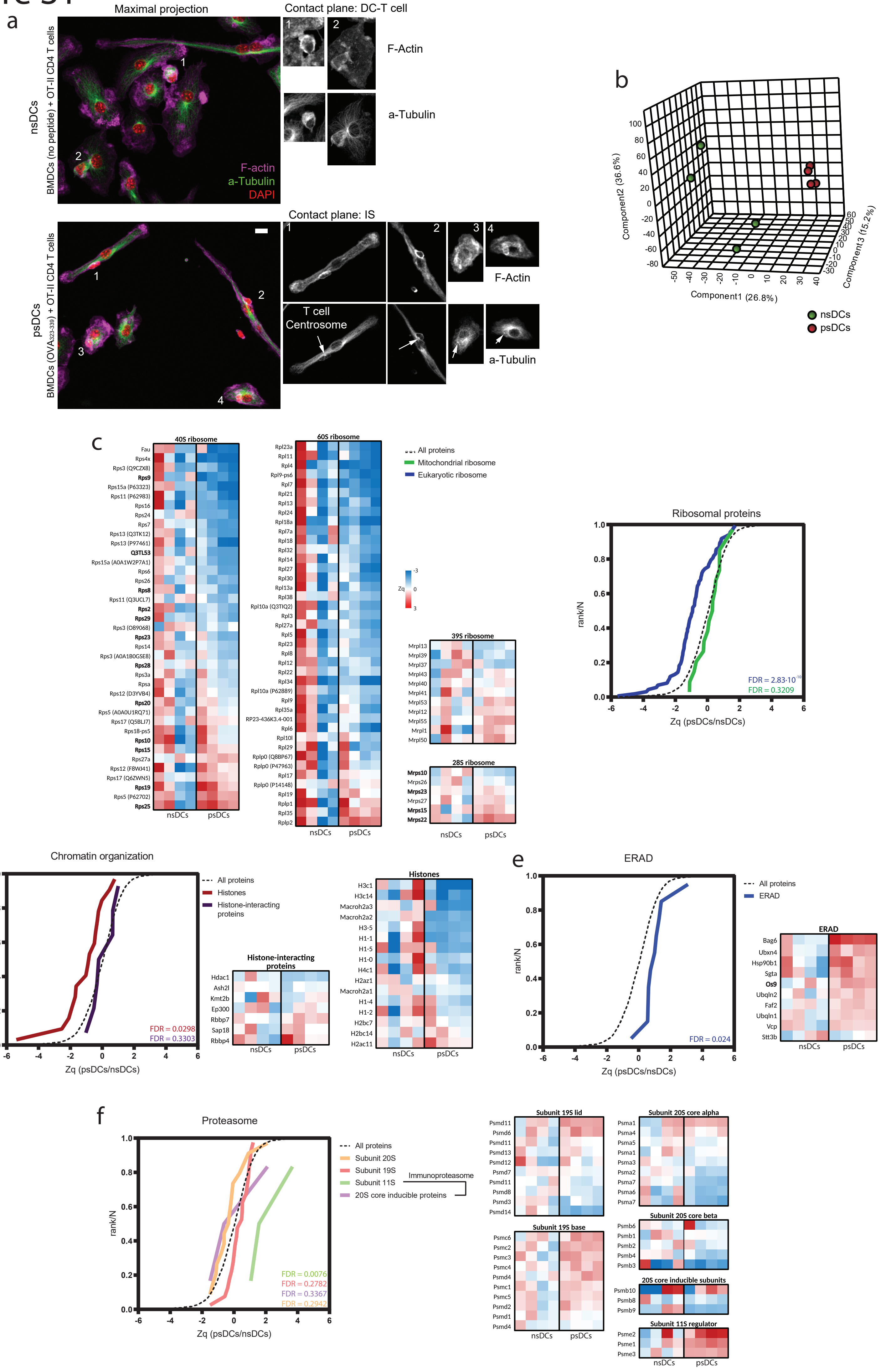
a
Maximal projection
Contact plane: DC-T cell
nsDCs
BMDCs (no peptide) + OT-II CD4 T cells
F-actin
a-Tubulin
DAPI
F-Actin
a-Tubulin
Contact plane: IS
psDCs
BMDCs (OVA323-339) + OT-II CD4 T cells
T cell
Centrosome
F-Actin
a-Tubulin
b
Component2 (36.6%)
Component1 (26.8%)
Component3 (15.2%)
nsDCs
psDCs
c
40S ribosome
60S ribosome
39S ribosome
28S ribosome
nsDCs
psDCs
Zq
All proteins
Mitochondrial ribosome
Eukaryotic ribosome
Ribosomal proteins
rank/N
Zq (psDCs/nsDCs)
FDR = 2.83·10^-10
FDR = 0.3209
d
Chromatin organization
All proteins
Histones
Histone-interacting proteins
FDR = 0.0298
FDR = 0.3303
Histones
e
ERAD
All proteins
ERAD
FDR = 0.024
f
Proteasome
All proteins
Subunit 20S
Subunit 19S
Subunit 11S
20S core inducible proteins
Immunoproteasome
FDR = 0.0076
FDR = 0.2782
FDR = 0.3367
FDR = 0.2942
Subunit 19S lid
Subunit 19S base
Subunit 20S core alpha
Subunit 20S core beta
20S core inducible subunits
Subunit 11S regulator

SUPPLEMENTARY FIGURE LEGENDS

Supplementary Figure 1. Dynamic changes in protein expression in psDCs vs. nsDCs. Related to Figure 1. (a) Left, a confocal fluorescence maximal projection image of LPS-activated BMDCs in coculture with T cells. BMDCs were pre-loaded (psDCs) or not (nsDCs) with $OVA_{323\text{-}339}$ peptide and conjugated with T cells for 3 h in glass-bottom chambers pre-coated with fibronectin. Cells were then fixed and processed for immunofluorescence. Magenta, F-actin (phalloidin); green, α-Tubulin; red, DAPI. Bar, 10 μm. Right, single confocal planes corresponding to the BMDC-T cell contact plane from the conjugates showed in the merge image. Single fluorescence channels for F-actin and α-tubulin are showed in grey-scale images. The centrosome is marked with a white arrow. (b) Partial least squares discriminant analysis (PLS-DA) of the psDC and nsDC biological replicates showing clustering of psDC samples performed with MetaboAnalyst 5.0. (c-f) Cumulative distribution of the protein abundance changes (Zq) showing the coordination in the behavior of proteins belonging to the selected functional categories compared to all proteins quantified in the experiment. Heatmaps showing the relative abundance changes (Zq) in all the nsDC and psDC biological replicates respect to the average value of the nsDC samples. Categories include (c) proteins belonging to the *Eukaryotic* (40S and 60S subunits) *and mitochondrial* (39S and 28S subunits) *ribosomes* or; (d) *Chromatin organization,* comparing histones and histone-interacting proteins; (e) the *ERAD pathway* related proteins; and (f) proteins belonging to the *Proteasome subunits* (11S, 19S, 20S and 20S core inducible proteins subunits).

Figure S2

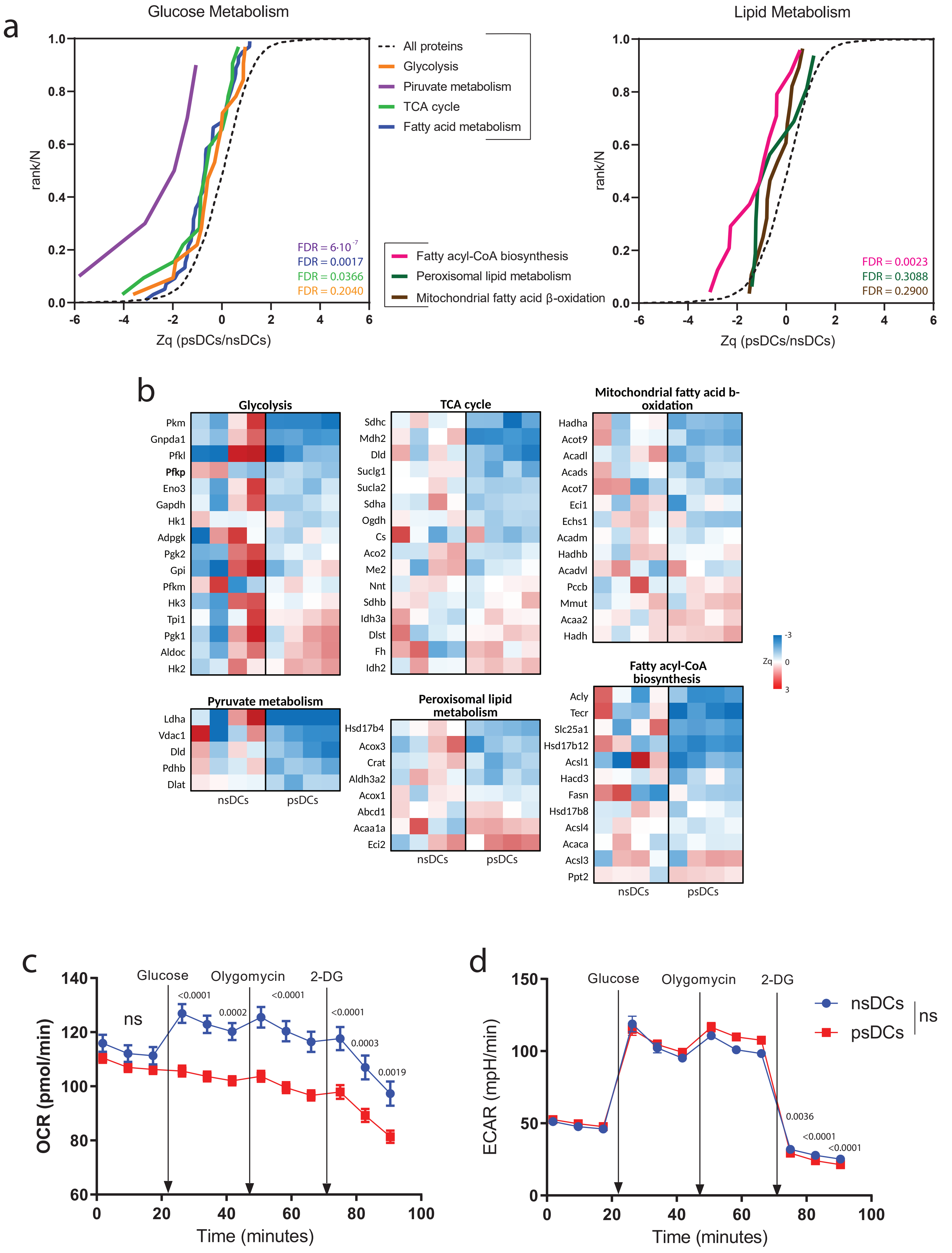
a
Glucose Metabolism
rank/N
Zq (psDCs/nsDCs)
All proteins
Glycolysis
Piruvate metabolism
TCA cycle
Fatty acid metabolism
FDR = 6·10-7
FDR = 0.0017
FDR = 0.0366
FDR = 0.2040
Lipid Metabolism
Fatty acyl-CoA biosynthesis
Peroxisomal lipid metabolism
Mitochondrial fatty acid β-oxidation
FDR = 0.0023
FDR = 0.3088
FDR = 0.2900
b
Glycolysis
Pkm
Gnpda1
Pfkl
Pfkp
Eno3
Gapdh
Hk1
Adpgk
Pgk2
Gpi
Pfkm
Hk3
Tpi1
Pgk1
Aldoc
Hk2
TCA cycle
Sdhc
Mdh2
Dld
Suclg1
Sucla2
Sdha
Ogdh
Cs
Aco2
Me2
Nnt
Sdhb
Idh3a
Dlst
Fh
Idh2
Mitochondrial fatty acid b-oxidation
Hadha
Acot9
Acadl
Acads
Acot7
Eci1
Echs1
Acadm
Hadhb
Acadvl
Pccb
Mmut
Acaa2
Hadh
Zq
-3
0
3
Pyruvate metabolism
Ldha
Vdac1
Dld
Pdhb
Dlat
nsDCs
psDCs
Peroxisomal lipid metabolism
Hsd17b4
Acox3
Crat
Aldh3a2
Acox1
Abcd1
Acaa1a
Eci2
nsDCs
psDCs
Fatty acyl-CoA biosynthesis
Acly
Tecr
Slc25a1
Hsd17b12
Acsl1
Hacd3
Fasn
Hsd17b8
Acsl4
Acaca
Acsl3
Ppt2
nsDCs
psDCs
c
Glucose
Olygomycin
2-DG
OCR (pmol/min)
ns
<0.0001
0.0002
<0.0001
<0.0001
0.0003
0.0019
Time (minutes)
d
Glucose
Olygomycin
2-DG
ECAR (mpH/min)
nsDCs
psDCs
ns
0.0036
<0.0001
<0.0001
Time (minutes)

Supplementary Figure 2. Coordinated protein changes reflect a change in metabolism in psDCs vs. nsDCs. Related to Figure 3. (a) Cumulative distribution of the protein abundance changes (Zq) corresponding to categories related to glucose (*Glycolysis*, *Pyruvate metabolism*, and *TCA cycle*; left) and lipid (*Fatty acyl-CoA biosynthesis*; *Peroxisomal lipid metabolism*, and *Mitochondrial fatty acid beta-oxidation*; right) metabolism. (b) Heatmaps showing the relative abundance changes (Zq) in all the nsDC and psDC biological replicates of the proteins belonging to the categories represented in A. (c) Mitochondrial respiration and (d) glycolytic activity obtained by Seahorse extracellular flux analysis of psDCs and nsDC during a glycolysis inhibition assay. For c-d $n = 8$ biological replicates with technical quintuplicates for each measure. Color code for c as in d. Data were analyzed in c and d using a two-way ANOVA. p-values are indicated, with ns for *p-value* > *0.05.* 2-DG: 2-Deoxy-D-glucose.

Figure S3

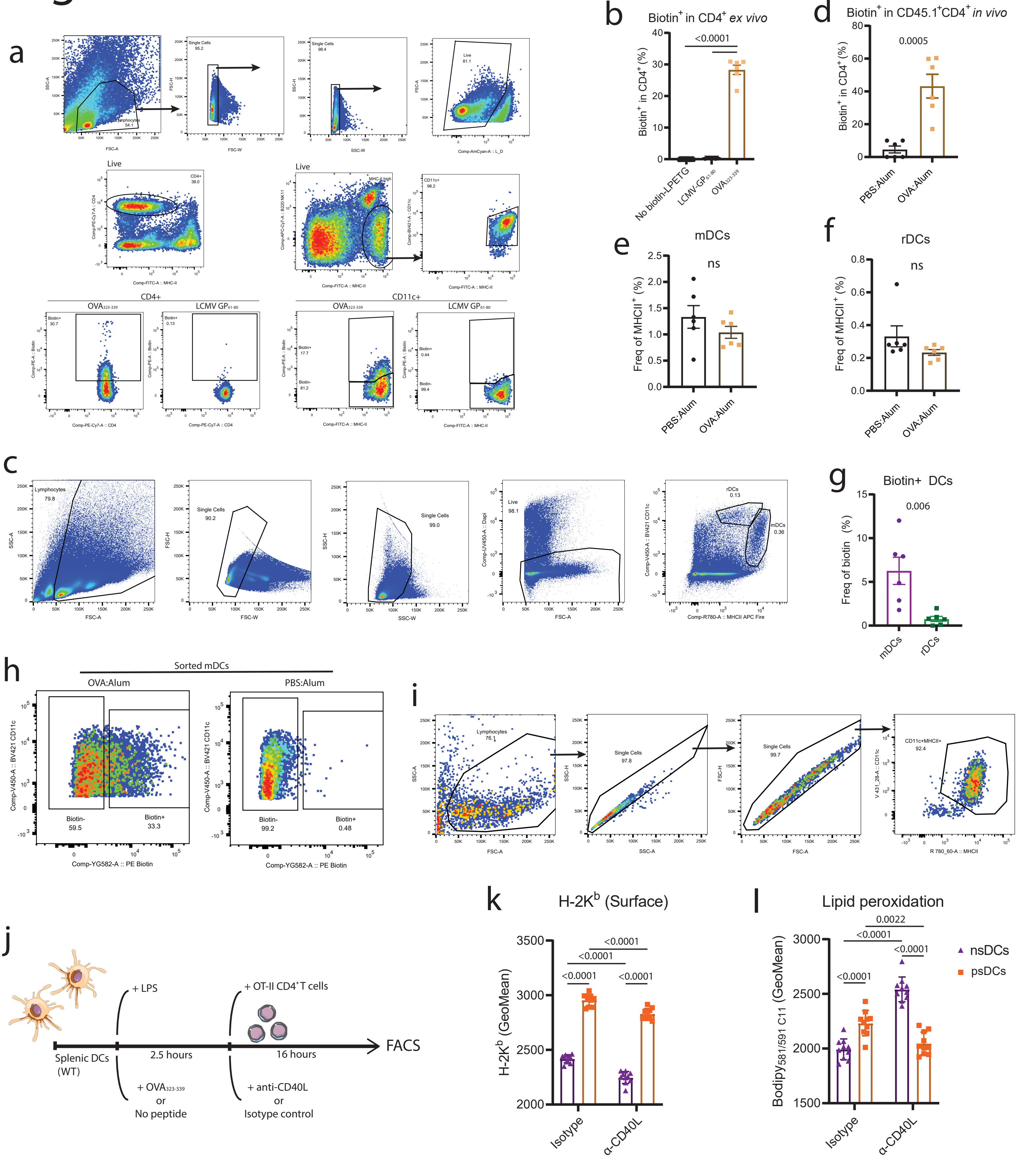
a
b
Biotin+ in CD4+ ex vivo
<0.0001
No biotin-LPETG
OVA323-339
d
Biotin+ in CD45.1+CD4+ in vivo
0.0005
PBS:Alum
OVA:Alum
e
mDCs
ns
Freq of MHCII+ (%)
f
rDCs
ns
c
g
Biotin+ DCs
0.006
Freq of biotin (%)
mDCs
rDCs
h
Sorted mDCs
OVA:Alum
PBS:Alum
Comp-YG582-A :: PE Biotin
i
j
+ LPS
+ OT-II CD4+T cells
FACS
Splenic DCs
(WT)
2.5 hours
16 hours
+ OVA323-339
or
No peptide
+ anti-CD40L
or
Isotype control
k
H-2Kb (Surface)
H-2Kb (GeoMean)
<0.0001
Isotype
α-CD40L
l
Lipid peroxidation
0.0022
<0.0001
Bodipy581/591 C11 (GeoMean)
nsDCs
psDCs

Supplementary Figure 3. Identification and analysis of biotin$^+$ populations in LIPSTIC experiments. Related to Figure 4. (a) FACS gating strategy for the *ex vivo* LIPSTIC experiment shown in Figure 3B-C. (b) Flow cytometry analysis of biotin$^+$ CD4$^+$ T cells in CD4$^+$CD45.1$^+$ cells (*Cd40lg^{SrtA}*) after coculture with DCs pulsed with the indicated peptide (LCMV $GP_{61\text{-}80}$ or $OVA_{323\text{-}339}$). No biotin-LPETG indicates pulsing with $OVA_{323\text{-}339}$ nut no addition of biotin-LPETG to the coculture. (c) Flow cytometry gating strategy used to identify mDCs in draining pLNs from immunized footpads in *in vivo* LIPSITC experiments. (d) Flow cytometry analysis of biotin$^+$ CD4$^+$ T cells in CD4$^+$CD45.1$^+$ cells (*Cd40lg^{Srta}*) extracted from popliteal lymph nodes (pLNs) from draining footpads immunized with the indicated formulation. FACS analysis of the proportions of (e) mDCs or (f) rDCs among live cells in pLNs of mice immunized with the indicated formulation. (g) Flow cytometry analysis of the proportions of biotin$^+$ cells within the indicated population of DCs. (g) FACS analysis of the frequency of biotin$^+$ mDCs in draining pLNs immunized with the indicated formulation. (g) Flow cytometry gating strategy to analyze the lipid peroxidation in sorted biotin$^+$ or biotin$^-$ mDCs shown in Figure 3H. (h) Sorting strategy to separate biotin$^+$ and biotin$^-$ within mDCs identified as in C. (i) Flow cytometry gating strategy for cells sorted as in H and stained with BODIPY™ 581/591 C11 for assessing lipid peroxidation in the CD11c$^+$MHCII$^+$ population. (j) *Ex vivo* experimental setup for K-L. Flow cytometry analysis of the geometric mean (GeoMean) of (k) H-2K^b or (l) lipid peroxidation in splenic DCs pulsed with $OVA_{323\text{-}339}$ (psDCs) or no peptide (nsDCs) generated in the presence of an anti-CD40L antibody or an isotype control. Data is representative of at least two independent experiments. For (b) and (d-g) $n = 6$. For (k-l) $n = 10$. Replicates are biological. Color code for (k) as in (l). Bar plots indicate mean ± SEM. Statistical significances are calculated with one-way ANOVA (b) or unpaired t-tests (d-g). p-values are indicated,

with ns for *p-value* > *0.05*. mDCs: migratory DCs. rDCs: resident DCs. pLNs: popliteal lymph nodes.

Figure S4

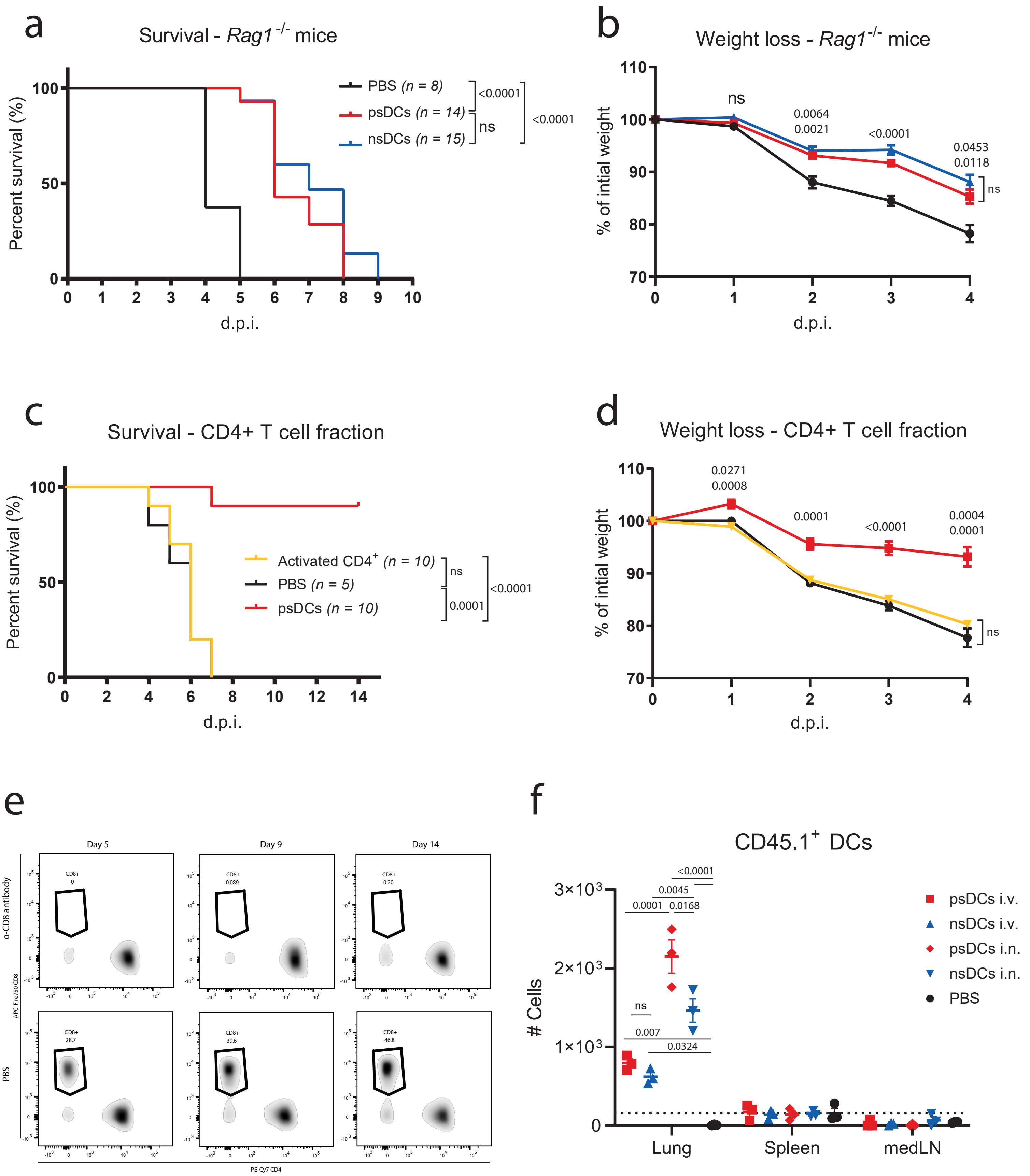
a
Survival - Rag1 -/- mice
Percent survival (%)
d.p.i.
PBS (n = 8)
psDCs (n = 14)
nsDCs (n = 15)
<0.0001
ns
<0.0001
b
Weight loss - Rag1 -/- mice
% of intial weight
d.p.i.
ns
0.0064
0.0021
<0.0001
0.0453
0.0118
ns
c
Survival - CD4+ T cell fraction
Percent survival (%)
d.p.i.
Activated CD4+ (n = 10)
PBS (n = 5)
psDCs (n = 10)
ns
0.0001
<0.0001
d
Weight loss - CD4+ T cell fraction
% of intial weight
d.p.i.
0.0271
0.0008
0.0001
<0.0001
0.0004
0.0001
ns
e
Day 5
Day 9
Day 14
α-CD8 antibody
PBS
APC-Fire750 CD8
PE-Cy7 CD4
CD8+
0
0.089
0.20
28.7
39.6
46.8
f
CD45.1+ DCs
Cells
Lung
Spleen
medLN
psDCs i.v.
nsDCs i.v.
psDCs i.n.
nsDCs i.n.
PBS
<0.0001
0.0045
0.0001
0.0168
ns
0.007
0.0324

Supplementary Figure 4. Controls of *Listeria monocytogenes* infection upon transfer of psDCs. Related to Figure 5. (a) Survival and (b) weight loss of *Rag1*$^{-/-}$ mice transferred with psDCs, nsDCs or PBS and then infected with a lethal dose of *Listeria monocytogenes*. Data are a pool of two independent experiments. For (b), *n* and color code as in (a). (c) Survival curves and (d) weight loss of mice that had been transferred $1 \cdot 10^6$ psDCs, $5 \cdot 10^4$ activated $CD4^+$ T cells purified from the psDC coculture or PBS. Data are a pool of two independent experiments. For (d), *n* and color code as in (c). (e) Proportions of $CD8^+$ T cells in the $CD3^+$ T cell population upon a single intraperitoneal inoculation of 50 µg of a depleting anti-CD8 antibody or PBS at 5, 9 or 14 days after inoculation. (f) Number of $CD45.1^+$ DCs (gated as CD45.1+CFSE+CD11c+) retrieved from the indicated organs 24 h after injection in $CD45.2^+$ WT mice (n = 3). The dot line indicated the limit of detection due to electronic noise of the FACS calculated as the mean of PBS values in the spleen samples. Replicates are biological (mice). In (a-d), statistical analysis of survival curves was performed with the Gehan-Breslow-Wilcoxon test. Weights were compared using a mixed-effect model with the Geisser-Greenhouse correction and Tukey's multiple comparison test. In (f) statistical analysis is an unpaired one-way ANOVA performed for each organ dataset. p-values are indicated, with ns for *p-value > 0.05.* D.p.i.: days post-infection.

Figure S5

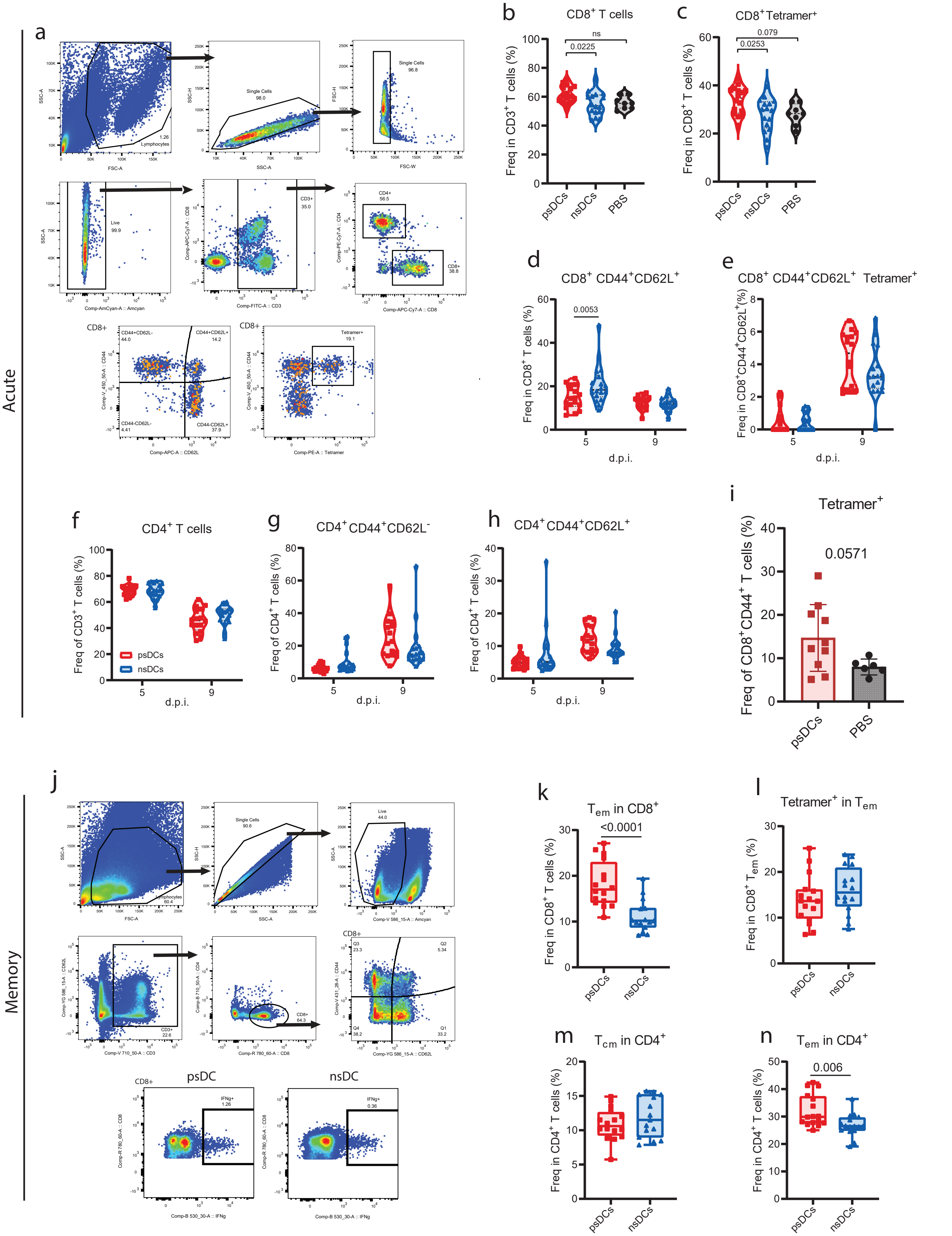

a
Acute
b
CD8+ T cells
Freq in CD3+ T cells (%)
ns
0.0225
psDCs
nsDCs
PBS
c
CD8+Tetramer+
Freq in CD8+ T cells (%)
0.079
0.0253
d
CD8+ CD44+CD62L+
Freq in CD8+ T cells (%)
0.0053
d.p.i.
e
CD8+ CD44+CD62L+ Tetramer+
Freq in CD8+CD44+CD62L+(%)
f
CD4+ T cells
Freq of CD3+ T cells (%)
g
CD4+CD44+CD62L-
Freq of CD4+ T cells (%)
h
CD4+CD44+CD62L+
i
Tetramer+
Freq of CD8+CD44+ T cells (%)
0.0571
j
Memory
psDC
nsDC
k
Tem in CD8+
<0.0001
Freq in CD8+ T cells (%)
l
Tetramer+ in Tem
Freq in CD8+ Tem (%)
m
Tcm in CD4+
Freq in CD4+ T cells (%)
n
Tem in CD4+
0.006

Supplementary Figure 5. Changes in other pathogen-specific acute and memory $CD8^+$ T cell populations upon influenza virus infection after transfer of psDCs. Related to Figure 6. (a) FACS gating strategy for peripheral blood cells. FACS analysis in peripheral blood at 9 after infection with an X31 influenza virus 24 h after injection of psDCs, nsDCs or PBS showing the frequency of (b) $CD8^+$ T cells within $CD3^+$ T cells, (c) $tetramer^+$ cells within $CD8^+$ T cells. FACS analysis in peripheral blood during acute infection at day 5 and 9 after infection showing: (d) the frequency of $CD44^+CD62L^+$ cells within $CD8^+$ T cells, (e) the frequency of tetramer+ cells within $CD44^+CD62L^+$ $CD8^+$ T cells, (f) proportion of $CD4^+$ within T cells, (g) frequency of $CD44^+CD62L^-$ within $CD4^+$ T cells, and (h) frequency of $CD44^+CD62L^+$ within $CD4^+$ T cells. Color code for d-h indicated in f. (i) Frequency of tetramer+ cells within CD8+CD44+ T cells in peripheral blood 9 days after infection of $Batf3^{-/-}$ mice that had been transferred with $5{\cdot}10^5$ psDCs or the same volume of PBS. (j) FACS gating strategy for splenocytes. FACS analysis in spleen of mice during the memory phase day 30 after infection showing: (k) the frequency of Tem ($CD44^+CD62L^-$) within $CD8^+$ T cells, (l) proportions of $tetramer^+$ cells within Tem $CD8^+$ T cells, (m) frequency of Tcm within $CD4^+$ T cells, and (n) frequency of Tem within $CD4^+$ T cells. All data are representative two independent experiments. For (b-c) $n = 16$ for psDC and nsDC and $n = 8$ for PBS, while for (d-h) $n=18$ for nsDC and $n = 17$ *at day 5 and* $n = 16$ at day 9 for psDC. For (k) and (m) $n=16$ for psDC and $n = 15$ for nsDC, while for (l) and (n) $n = 16$. For (i) $n = 10$ for psDC and $n = 6$ for PBS. Replicates are biological (mice). Statistical analyses are result of one-way ANOVA (b-c), a mixed-effect model with and Sidák's multiple comparison test (d-h) or unpaired t-test (i and k-n). MFI: mean fluorescence intensity. p-values are indicated, with ns for $p\text{-value} > 0.05$. D.p.i.: days post-infection.

Figure S6

a

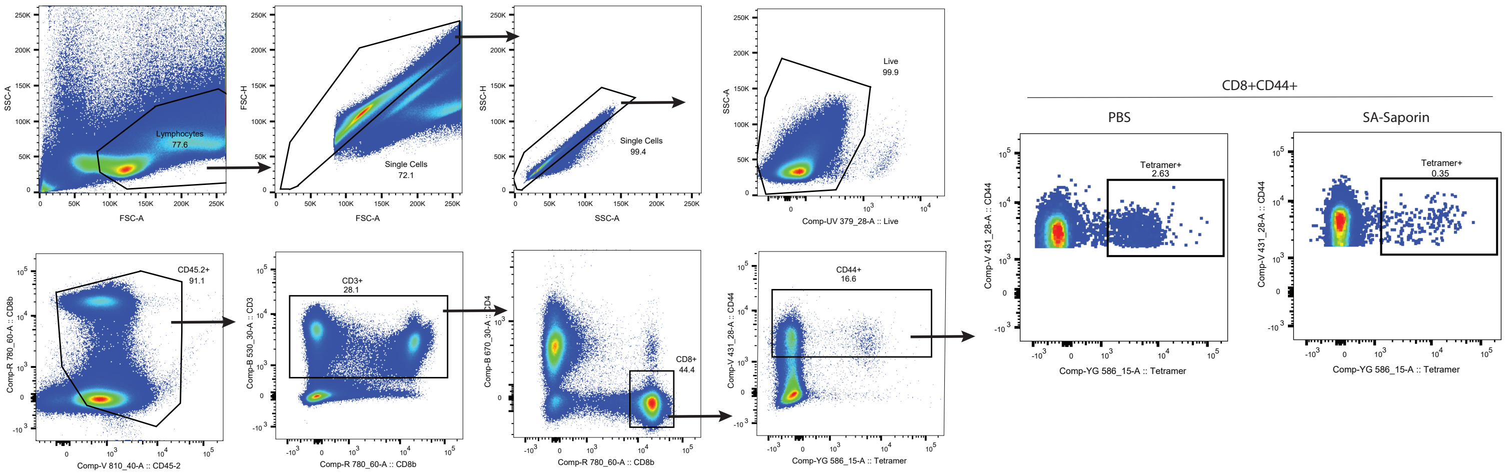
Lymphocytes 77.6
FSC-A
SSC-A
FSC-H
Single Cells 72.1
SSC-H
Single Cells 99.4
Live 99.9
Comp-UV 379_28-A :: Live
CD45.2+ 91.1
Comp-V 810_40-A :: CD45-2
Comp-R 780_60-A :: CD8b
CD3+ 28.1
Comp-B 530_30-A :: CD3
Comp-B 670_30-A :: CD4
CD8+ 44.4
CD44+ 16.6
Comp-V 431_28-A :: CD44
Comp-YG 586_15-A :: Tetramer
CD8+CD44+
PBS
SA-Saporin
Tetramer+ 2.63
Tetramer+ 0.35

b

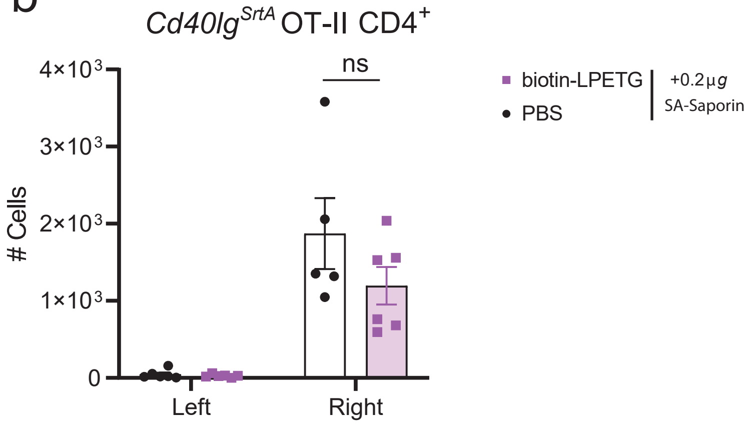
Cd40lg^SrtA OT-II CD4+
Cells
4×10^3
3×10^3
2×10^3
1×10^3
0
ns
Left
Right
biotin-LPETG
PBS
+0.2μg SA-Saporin

Supplementary Figure 6. Identification of SIINFEKL-specific CD8$^+$ T cell upon immunization. Related to Figure 7. (a) Flow cytometry gating strategy of popliteal lymph nodes identifying SIINFEKL-specific CD8$^+$ T cells upon psDC depletion shown in Figure 6. (b) Counts of CD40L-SrtA OT-II CD4$^+$ T cells (gated as CD45.1$^+$CD4$^+$ T cells) in draining popliteal lymph nodes from the immunized (right) or contralateral (left) side 8 days after immunization. The counts represent the cells contained in one full pLN. Data are representative of two independent experiments. $N = 6$ animals. Bar plots indicate mean ± SEM. Statistical tests are unpaired two-way ANOVA. Data is representative two independent experiments. For ns, *p-value > 0.05.*
