# Supplementary material for: Immune synapse formation promotes lipid peroxidation and MHC-I upregulation in licensed dendritic cells for efficient priming of CD8+ T cells
Source: Nat Commun. 2023 Oct 25;14:6772. doi: 10.1038/s41467-023-42480-3 (PMC10600134; doi:10.1038/s41467-023-42480-3)
Supplement: Supplementary file 3 — Description of Additional Supplementary Files [file 41467_2023_42480_MOESM3_ESM.pdf]

Description of Additional Supplementary Files

Supplementary Data 1. Protein abundance changes in postsynaptic DCs (psDCs) in comparison to nonsynaptic DCs (nsDCs). Related to Figure 1. Results are reported as Zq values, which are log2-fold changes expressed in units of standard deviation for each individual biological replicate and for the integration data in the comparative of psDC vs. nsDC samples. N indicates the number of quantified peptides for each protein.

Supplementary Data 2. **Functional category abundance changes in postsynaptic DCs (psDCs) in comparison to nonsynaptic DCs (nsDCs).** Related to Figure 1. Results are reported as Zc values, which are log2-fold changes expressed in units of standard deviation each individual biological replicate and for the integration data in the comparative of psDC vs. nsDC samples. N indicates the number of quantified proteins for each functional category.

Supplementary Data 3. IPA analysis of psDCs vs nsDCs. Related to Figure 3. Diseases and functions categories predicted by Ingenuity Pathway Analysis (IPA). Results are reported including the p-value, activation state prediction, activation z-score, names and number of the molecules included per category.
