# Supplementary material for: Immune synapse formation promotes lipid peroxidation and MHC-I upregulation in licensed dendritic cells for efficient priming of CD8+ T cells
Source: Nat Commun. 2023 Oct 25;14:6772. doi: 10.1038/s41467-023-42480-3 (PMC10600134; doi:10.1038/s41467-023-42480-3)
Supplement: Supplementary file 7 — Reporting Summary [file 41467_2023_42480_MOESM7_ESM.pdf]

nature portfolio

Corresponding author(s): Francisco Sánchez Madrid

Last updated by author(s): Sep 21, 2023

Reporting Summary

Nature Portfolio wishes to improve the reproducibility of the work that we publish. This form provides structure for consistency and transparency in reporting. For further information on Nature Portfolio policies, see our Editorial Policies and the Editorial Policy Checklist.

Statistics

For all statistical analyses, confirm that the following items are present in the figure legend, table legend, main text, or Methods section.

n/a	Confirmed	
[ ]	[x]	The exact sample size (*n*) for each experimental group/condition, given as a discrete number and unit of measurement
[ ]	[x]	A statement on whether measurements were taken from distinct samples or whether the same sample was measured repeatedly
[ ]	[x]	The statistical test(s) used AND whether they are one- or two-sided<br>*Only common tests should be described solely by name; describe more complex techniques in the Methods section.*
[x]	[ ]	A description of all covariates tested
[ ]	[x]	A description of any assumptions or corrections, such as tests of normality and adjustment for multiple comparisons
[ ]	[x]	A full description of the statistical parameters including central tendency (e.g. means) or other basic estimates (e.g. regression coefficient) AND variation (e.g. standard deviation) or associated estimates of uncertainty (e.g. confidence intervals)
[ ]	[x]	For null hypothesis testing, the test statistic (e.g. *F*, *t*, *r*) with confidence intervals, effect sizes, degrees of freedom and *P* value noted<br>*Give P values as exact values whenever suitable.*
[x]	[ ]	For Bayesian analysis, information on the choice of priors and Markov chain Monte Carlo settings
[x]	[ ]	For hierarchical and complex designs, identification of the appropriate level for tests and full reporting of outcomes
[x]	[ ]	Estimates of effect sizes (e.g. Cohen's *d*, Pearson's *r*), indicating how they were calculated

Our web collection on statistics for biologists contains articles on many of the points above.

Software and code

Policy information about availability of computer code

Data collection	No software was used for data collection
Data analysis	Statistical analysis were performed with GraphPad Prism 8.0 (GraphPad Software, 758 San Diego, CA, USA).<br>Analysis of protein abundance was performed using the SanXoT package available at cnic-proteomics.github.io/iSanXoT/ [Reference: Trevisan-Herraz, M. et al. SanXoT: a modular and versatile package for the quantitative analysis of high-throughput proteomics experiments. Bioinformatics 35, 1594–1596 (2019)].<br>Partial least squares discriminant analysis (PLS-DA) was calculated and represented using the MetaboAnalyst 5.0 software

For manuscripts utilizing custom algorithms or software that are central to the research but not yet described in published literature, software must be made available to editors and reviewers. We strongly encourage code deposition in a community repository (e.g. GitHub). See the Nature Portfolio guidelines for submitting code & software for further information.

Data

Policy information about availability of data

All manuscripts must include a data availability statement. This statement should provide the following information, where applicable:

- Accession codes, unique identifiers, or web links for publicly available datasets
- A description of any restrictions on data availability
- For clinical datasets or third party data, please ensure that the statement adheres to our policy

The mass spectrometry proteomics data have been deposited to the ProteomeXchange Consortium via the PRIDE partner repository with the dataset identifier PXD039035 and is publicly available as of the date of publication (https://proteomecentral.proteomexchange.org/cgi/GetDataset?ID=PXD039035). This paper does not report original code.

Research involving human participants, their data, or biological material

Policy information about studies with human participants or human data. See also policy information about sex, gender (identity/presentation), and sexual orientation and race, ethnicity and racism.

Reporting on sex and gender	n/a
Reporting on race, ethnicity, or other socially relevant groupings	n/a
Population characteristics	n/a
Recruitment	n/a
Ethics oversight	n/a

Note that full information on the approval of the study protocol must also be provided in the manuscript.

Field-specific reporting

Please select the one below that is the best fit for your research. If you are not sure, read the appropriate sections before making your selection.

☒ Life sciences ☐ Behavioural & social sciences ☐ Ecological, evolutionary & environmental sciences

For a reference copy of the document with all sections, see nature.com/documents/nr-reporting-summary-flat.pdf

Life sciences study design

All studies must disclose on these points even when the disclosure is negative.

Sample size	Sample size was not predetermined for in vivo experiments, and was limited to the availability of mice for each experimental group or the amount of cells generated to be transferred into the animals. In vitro experiments were mostly performed using a n = 8, as our bone marrow donor mice were in groups of 8 animals. Experiments were performed multiple times and conclusions were considered valid if results were reproducible between experiments.
Data exclusions	In figure S6B, an identified outlayer with the ROUT method (Q = 1%) was removed in the PBS-Right group (value ca. 10 times the mean value of the sample group). No other exclusions were performed.
Replication	All data presented in this study were replicated at least in two independent experiments except for the proteomic study due to economic reasons and personnel and time available for analysis. No differences were observed between replications.
Randomization	Mice were randomly allocated to experimental groups, maintaining the homogeneity in terms of sex and age between experimental groups.
Blinding	Investigators were blinded to group allocation or during data collection but not analysis. This is due to a lack of personnel and resources.

Reporting for specific materials, systems and methods

We require information from authors about some types of materials, experimental systems and methods used in many studies. Here, indicate whether each material, system or method listed is relevant to your study. If you are not sure if a list item applies to your research, read the appropriate section before selecting a response.

Materials & experimental systems

n/a	Involved in the study
	x Antibodies
x	Eukaryotic cell lines
x	Palaeontology and archaeology
	x Animals and other organisms
x	Clinical data
x	Dual use research of concern
x	Plants

Methods

n/a	Involved in the study
x	ChIP-seq
	x Flow cytometry
x	MRI-based neuroimaging

Antibodies

Antibodies used

anti-mouse CD16/CD32, clone 2.4G2 Tonbo Biosciences Cat# 70-0161; RRID: AB_2621487
InVivoMAb anti-mouse CD8β (Lyt 3.2) BioXCell Cat# BE0223; RRID: AB_2687706
Alexa Fluor® 647 anti-mouse H-2Kb antibody BioLegend Cat# 116511; RRID: AB_492918
Alexa Fluor® 647 anti-mouse IL-2 Antibody BioLegend Cat# 503814; RRID: AB_493517
APC/Fire™ 750 anti-mouse CD8a Antibody BioLegend Cat# 100766; RRID: AB_2572113
PE anti-mouse CD36 Antibody BioLegend Cat# 102605; RRID: AB_389348
APC/Fire™ 750 anti-mouse I-A/I-E Antibody BioLegend Cat# 107652; RRID: AB_2616729
Brilliant Violet 785™ anti-mouse CD45.2 Antibody BioLegend Cat# 109839; RRID: AB_2562604
APC/Cyanine7 anti-mouse/human CD45R/B220 Antibody BioLegend Cat# 103223; RRID: AB_313006
APC/Cyanine7 anti-mouse NK-1.1 Antibody BioLegend Cat# 108723; RRID: AB_830870
FITC Rat Anti-Mouse I-A/I-E BD Biosciences Cat# 553623; RRID: AB_394958
BV421 Armenian Hamster Anti-Mouse CD11c BD Biosciences Cat# 565451; RRID: AB_2744278
BV711 Hamster Anti-Mouse CD3e BD Biosciences Cat# 563123; RRID: AB_2687954
FITC Rat Anti-Mouse CD62L BD Biosciences Cat# 553150; RRID: AB_394665
PE Rat Anti-Mouse CD62L BD Biosciences Cat# 553151; RRID: AB_394666
PerCP-Cyanine5.5 Anti-Mouse CD45.1 (A20) antibody Tonbo Biosciences Cat# 65-0453; RRID: AB_2621893
PE-Cyanine7 Anti-Mouse CD4 (GK1.5) Antibody Tonbo Biosciences Cat# 60-0041; RRID: AB_2621828
FITC Anti-Mouse CD3e (145-2C11) Antibody Tonbo Biosciences Cat# 35-0031; RRID: AB_2621659
APC Anti-Mouse CD62L (L-Selectin) (MEL-14) antibody Tonbo Biosciences Cat# 20-0621; RRID: AB_2621582
violetFluor 450 Anti-Mouse CD4 (GK1.5) Antibody Tonbo Biosciences Cat# 75-0041; RRID: AB_2621927
APC Anti-Mouse CD4 (RM4-5) Antibody Tonbo Biosciences Cat# 20-0042; RRID: AB_2621544
PerCP Anti-Mouse CD4 (RM4-5) Tonbo Biosciences
Rat Anti-CD25 Monoclonal Antibody, Biotin Conjugated, Clone 7D4 BD Biosciences Cat# 553070; RRID: AB_394602
CD8b Monoclonal Antibody (eBioH35-17.2 (H35-17.2)), APC-eFluor 780, eBioscience Thermo Fisher Scientific Cat# 47-0083-82; RRID: AB_2573943
IFN gamma Monoclonal Antibody (XMG1.2), FITC, eBioscience Thermo Fisher Scientific Cat# 11-7311-81; RRID: AB_465411
BV421 Streptavidin BD Biosciences Cat# 563259 ; RRID: AB_2869475
Rat Anti-CD62L Monoclonal Antibody, Phycoerythrin Conjugated, Clone MEL-14, BD Biosciences Cat# 553151, RRID:AB_394666
Ultra-LEAF(TM) Purified anti-mouse CD154, BioLegend Cat# 106517, RRID:AB_2813947
Ultra-LEAF™ Purified Armenian Hamster IgG Isotype Ctrl Antibody, BioLegend Cat# 400959, RRID:AB_11203531
Monoclonal Anti-alpha-Tubulin-FITC antibody produced in mouse, Sigma-Aldrich Cat# F2168, RRID:AB_476967

Validation

All antibodies used were commercially validated. The validation can be consulted in the manufacturer's webpage.

Animals and other research organisms

Policy information about studies involving animals; ARRIVE guidelines recommended for reporting animal research, and Sex and Gender in Research

Laboratory animals

Mouse strains included in this study comprise C57BL/6JOlaHsd (or C57BL/6) wild-type mice; B6-SJL (Ptprca Pepcb/BoyJ) expressing CD45.1 allele; TCR transgenic OT-II mice (B6.Cg-Tg(TcraTcrb)425Cbn/J) and TCR transgenic OT-I mice (C57BL/6-Tg(TcraTcrb)1100Mjb/J) both mated with B6-SJL (Ptprca Pepcb/BoyJ); Rag1-/- mice (B6.129S7-Rag1tm1Mom/J); β2m-/- mice (B6.129P2-B2mtm1Unc/DcrJ) from The Jackson Laboratories. Cd40G5 and Cd40lgSrta (LIPSTIC mice) were kindly provided by Dr. Giulia Pasqual (University of Padova, Italy). Batf3-/- mice (B6.129S(C)-Batf3tm1Kmm/J) were kindly provided by Dr. David Sancho (CNIC). Mice were kept on dark/light cycle 12:12, ambient temperature 22ºC ± 2, and humidity 55% ± 10. Female 8 to 12-week-old mice were used unless otherwise indicated. In the case of LIPSTIC mice, both males and females were used.

Wild animals

No wild animals have been used.

Reporting on sex

For BMDCs cultures, bona marrows from female 8 to 12-week-old mice were used. OT-II CD4+ T cells were extracted from both male and female mice. Infeciton experiments were performed on female mice. For LIPSTIC mice, both males and females were used. Littermates were randomly assigned to experimental groups.

Field-collected samples	No field-collected samples were used.
Ethics oversight	Animal experiments were approved by the local Ethics Committee for Basic research at the CNIC Ethical Committee for Animal Welfare and the Organo Encargado del Bienestar Animal (OEBA) del Gabinete Veterinario de la Universidad Autonoma de Madrid (UAM) and are in agreement with EU Directive 86/609/EEC and Recommendation 2007/526/EC regarding the protection of animals used for experimental and other scientific purposes, enforced in Spanish law under Real Decreto 53/2013. Additionally, experiments were approved by Italian Ministry of Health (Authorization n. 994/2020-PR).

Note that full information on the approval of the study protocol must also be provided in the manuscript.

Flow Cytometry

Plots

Confirm that:

- [x] The axis labels state the marker and fluorochrome used (e.g. CD4-FITC).
- [x] The axis scales are clearly visible. Include numbers along axes only for bottom left plot of group (a 'group' is an analysis of identical markers).
- [x] All plots are contour plots with outliers or pseudocolor plots.
- [x] A numerical value for number of cells or percentage (with statistics) is provided.

Methodology

Sample preparation	Spleens and lymph nodes were perfused with Liberase TL (250 µg/mL) and DNAse I (10 µg/mL) and incubated at 37 °C for 20 min in HBSS (Lonza), then grinded on 70 µm mesh prior to lysing of erythrocytes with ACK buffer (Lonza) in the case of spleens.<br>BMDCs:CD4+ T cell cocultures were detached with phosphate-buffered saline (PBS), EDTA (5 mM), and bovine serum albumin (BSA) 0.5% (PBE).<br>Flow cytometry samples were first stained for cell viability according to manufacturer's instructions and Fc receptors were blocked using Anti-mouse CD16/CD32 (Fc Shield) for 15 min in PBS at 4 °C. Then, samples were resuspended in antibody cocktails for a minimum of 20 min in ice-cold PBE. For intracellular staining, cells were then fixed, permeabilized and stained with the BD Cytofix/Cytoperm kit following manufacturer's instructions. Antibodies and tetramers used are included in the key resources table.
Instrument	Samples were acquired using an LSR Fortessa or BD FACSymphonyTM (BD Biosciences).
Software	Data were acquired with BD FACSDiva software v9.1 (BD Biosciences) and analyzed with FlowJo software version 10 (TreeStar)
Cell population abundance	Cell population abundances and counts are provided in the graphs and gating strategies. Sorted cells from figure 3H were submitted to FACS analysis: shown in figure S3I.
Gating strategy	Complete gating strategies are provided in supplementary figures except BMDCs:CD4+ T cell cocultures, in which the analyzed DC population was gated via FSC-A/SSC-A for size and complexity, SSC-A/SSC-H for singlets, SSC-A/Live Dead marker for live cells and CD11c/CD4 for CD11c+ cells.

- [x] Tick this box to confirm that a figure exemplifying the gating strategy is provided in the Supplementary Information.
